# Spectral photon-counting CT in first-pass myocardial perfusion imaging for very high-risk patients: a comparison with dual-energy CT

**DOI:** 10.1186/s41747-025-00624-8

**Published:** 2025-09-20

**Authors:** Guillaume Fahrni, Salim Si-Mohamed, Rafael Wiemker, David C. Rotzinger, Angèle Houmeau, Cyril Prieur, Philippe Douek, Sara Boccalini

**Affiliations:** 1 Cardiothoracic and Vascular Division, Department of Diagnostic and Interventional Radiology, Lausanne University Hospital and University of Lausanne, Lausanne, Switzerland; 2 Radiology Department, Hospices Civils de Lyon, Lyon, France; 3 INSA-Lyon, University of Lyon, University Claude-Bernard Lyon 1, UJM-Saint-Étienne, CNRS, Inserm, CREATIS UMR 5220, Lyon, France; 4 Philips Innovative Technologies, Hamburg, Germany; 5 Department of Cardiology, Hôpital Louis Pradel, Hospices Civils de Lyon, Bron, France

**Keywords:** Computed tomography angiography, Dual energy computed tomography, Myocardial perfusion imaging, Coronary stenosis, Fractional flow reserve

## Abstract

**Background:**

Spectral photon-counting computed tomography (SPCCT) outperformed dual-energy computed tomography (DECT) for coronary artery stenosis assessment. However, data about myocardial perfusion imaging (MPI) is lacking. This feasibility study aimed to evaluate and compare the diagnostic performance of SPCCT and DECT for rest MPI in patients with hemodynamically significant coronary stenoses, using invasive coronary angiography (ICA) and invasive fractional flow reserve (FFR) as reference standards.

**Materials and methods:**

Eighteen very-high-risk patients with hemodynamically significant coronary stenoses at ICA underwent both dual-layer DECT and SPCCT coronary CT within three days. The sensitivity, specificity, and accuracy of MPI in detecting myocardial hypoperfusion were assessed. Quantitative attenuation differences between normal and hypoperfused myocardial segments were compared for both modalities. Interobserver variability was assessed with a weighted kappa analysis.

**Results:**

SPCCT demonstrated comparable overall performance to DECT for MPI, with an overall sensitivity, specificity, and accuracy of 73.3%, 79.2%, and 76.9%, respectively, *versus* 73.3%, 75%, and 74.4% for DECT. SPCCT outperformed DECT in the left anterior descending artery territory, achieving a sensitivity of 87.5%, specificity of 100%, and accuracy of 90%, *versus* 62.5%, 50%, and 60% for DECT. For each CT system, attenuation analysis revealed differences between normal and hypoperfused segments, with mean differences of 17.9 HU for DECT and 15.8 HU for SPCCT (*p* < 0.05). Inter-reader agreement was higher for SPCCT (κ = 0.86) compared to DECT (κ = 0.62).

**Conclusion:**

SPCCT and DECT provided similar diagnostic performance for rest MPI in a very-high-risk patient cohort, demonstrating comparable effectiveness in detecting the effects of hemodynamically significant coronary stenosis.

**Relevance statement:**

Hemodynamically significant stenosis in very-high-risk patients results in myocardial hypoperfused areas at rest that can be detected equally well with dual-layer CT and spectral photon counting CT, albeit with better reproducibility for the latter.

**Key Points:**

- SPCCT and DECT showed comparable performance for MPI at rest in very-high-risk patients.
- The differences between normal and hypoperfused segments were of 17 HU and 16 HU on conventional images for DECT and SPCCT.
- SPCCT showed higher interobserver agreement compared to DECT, suggesting improved reproducibility.

**Graphical Abstract:**

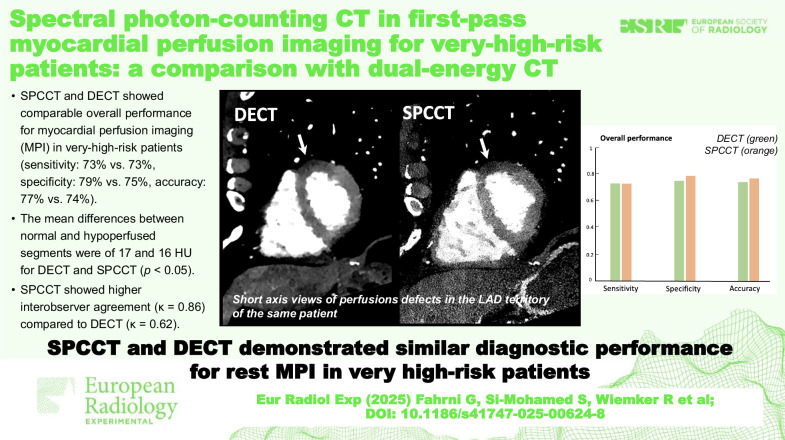

## Introduction

Coronary computed tomography angiography (CCTA) is a widely used non-invasive technique for assessing coronary artery disease, known for its reliable negative and positive predictive values [1]. Recent ESC Guidelines particularly recommend CCTA for individuals at low to intermediate risk of coronary artery disease [2]. In contrast, patients with a high pre-test risk may benefit from an assessment of functional parameters, while invasive coronary angiography (ICA) is generally recommended for those at very high risk. In this context, first-pass rest myocardial perfusion imaging (MPI) can be performed during the same CT examination, providing derivative perfusion information about normal and hypoperfused myocardial territories [3, 4]. A rest MPI assessment differs from a dedicated MPI assessment performed under stress conditions, in that only severe stenoses in the order of 70–80% induce a hypoperfusion detectable at rest [5, 6]. Using invasive fractional flow reserve (FFR) with a value of < 0.8 as a gold standard reference for hemodynamically significant coronary stenosis, the combined use of CCTA and rest MPI has shown the potential to reduce the rate of false-positive stenosis with conventional CT [7]. Dual-energy computed tomography (DECT) enables further possibilities for myocardium analysis. This modality is based on different technologies of source or detector, such as fast kilovoltage switching, dual source, double rotation, and split beam or dual-layer detector [8]. With DECT, separation between low and high-energy x-ray photons results in several new types of image reconstructions, such as virtual monoenergetic images and iodine maps. These specific maps possess interesting features such as enhanced iodine attenuation and iodine quantification [9–11], helping improve contrast resolution in tissues such as the myocardium.

Recent technological advances have led to the introduction of spectral photon counting computed tomography (SPCCT), with the potential to advance the field of spectral imaging [12–14]. The previously used indirect-conversion energy integrating detector (EID) is replaced with a direct-conversion photon counting detector (PCD) that brings major improvements in cardiovascular imaging [15, 16]. Indeed, SPCCT has already shown significant advancement for CCTA in humans, compared to conventional or DECT imaging, mainly due to its improved spatial resolution and blooming artefact reduction [17–19]. However, its impact on MPI, compared with DECT, has not yet been established [20]. Notably, no studies have specifically assessed its performance in a population with very-high cardiovascular risk, which could particularly benefit from the technological advancements of SPCCT. Given its higher spatial resolution, reduced electronic noise, as well as purported enhanced spectral capabilities, and improved iodine quantification, SPCCT may overcome several known limitations of DECT for MPI. We therefore hypothesised that SPCCT would perform at least, as well as DECT for the detection of rest myocardial hypoperfusion in patients with hemodynamically significant coronary stenoses.

Therefore, the purpose of this study was to evaluate the performance of MPI between SPCCT and dual-layer DECT, in a very high-risk population of patients with hemodynamically significant coronary artery stenoses.

## Materials and methods

### Study design

This prospective observational monocentric feasibility study was conducted in a tertiary care university hospital (Hôpital Louis Pradel, Hospices Civils de Lyon, Lyon, France). The study was approved by the local ethics committee (approval number: 2019-A02945–52). Written informed consent was provided by all patients.

### Population

**Fig. 1:**
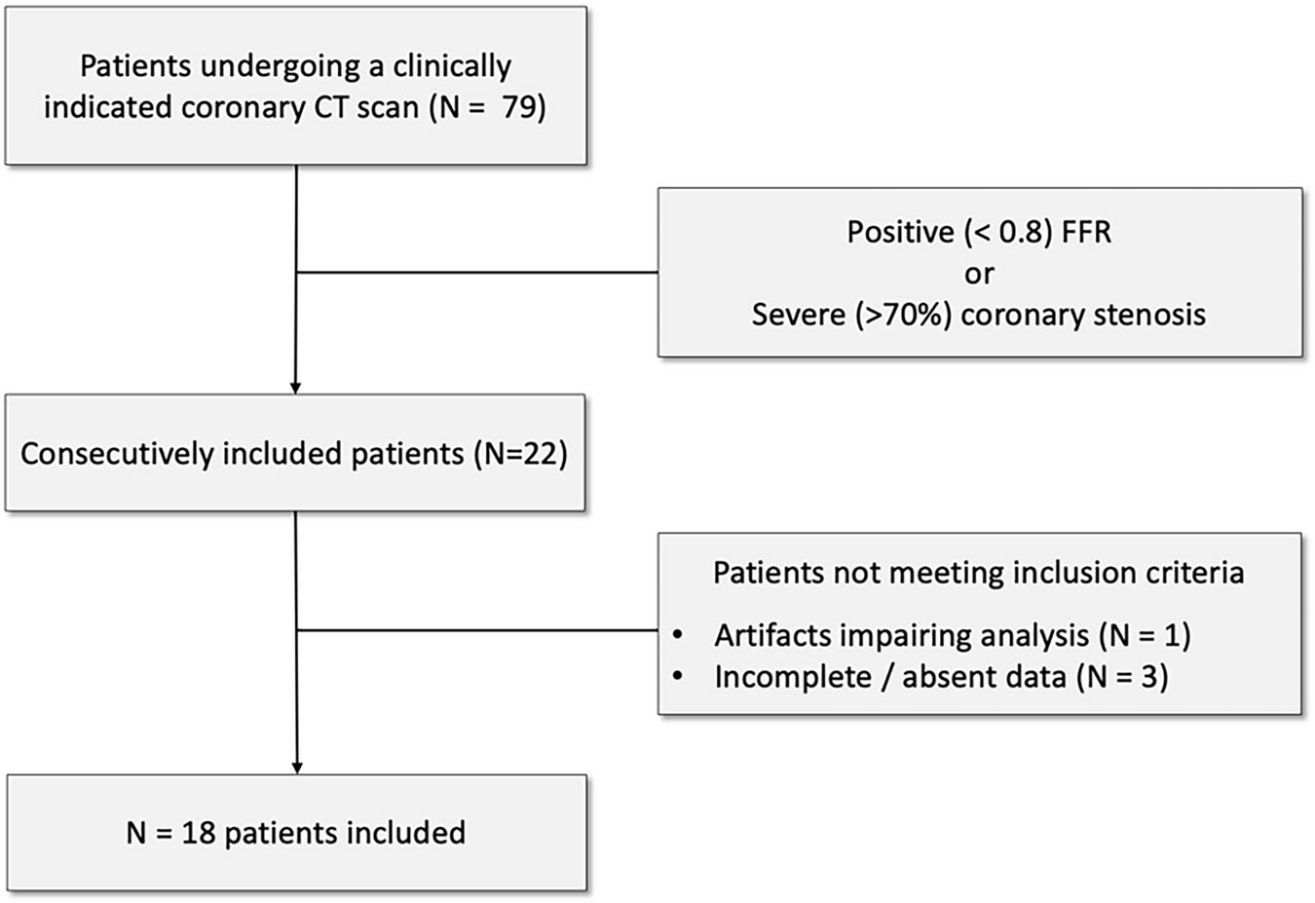
Study flowchart

Consecutive patients referred for CCTA for suspicion of coronary artery disease or with known chronic coronary disease necessitating a pre-interventional assessment from February 2021 to April 2022 were further evaluated for inclusion (*N* = 79). Thereafter, patients who underwent an ICA with FFR study, were consecutively included (*N* = 22). Hemodynamically significant stenosis was defined as either a confirmed FFR-positive stenosis (FFR < 0.8) or a severe (> 70%) coronary stenosis. All three exams (SPCCT, DECT coronary CT and ICA) were performed within 3 days. Patients with artefacts impeding the analysis of most segments (*N* = 1) or with incomplete data (*N* = 3) were excluded from the analysis, leaving a total of 18 included cases (Fig. 1). All patients were classified as very-high risk category according to the 2021 ESC Guidelines on cardiovascular disease prevention in clinical practice [21].

### CT acquisitions

Patients underwent CCTA on both DECT and SPCCT systems within 3 days following diagnostic ICA. DECT systems were two commercially available dual-layer spectral CTs (IQon spectral CT and CT7500; Philips Healthcare). The SPCCT system was a clinical prototype, with a large field of view (FOV = 500 mm in-plane). It is equipped with a single-layer PCD made of cadmium zinc telluride with a pixel pitch of 275 × 275 μm^2^ at isocentre [22, 23]. Patients were placed in the supine position, arms above the head. Intravenous beta-blockers (esmolol chlorohydrate, Esmocard) were used, when necessary, with a ≤ 65 bpm target heart rate. A dose of sublingual nitroglycerin spray (Natispray; Teofarma SRL, Italy) was administered to all patients without contraindication. A bolus of 65–75 mL of Iomeprol (400 mg/mL; Iomeron®, Bracco) at 4 mL/s was used, depending on the patient’s weight (threshold of 80 kg), followed by a saline flush. A retrospective ECG-gating helical mode was used for both systems, in the arterial phase. The detailed scanning parameters were: tube potential: 120 kVp; tube current: 255 mAs; automatic exposure control with DoseRight index of 28 for DECT; rotation time: 0.27 for DECT and 0.33 for SPCCT; field-of-view: 220 mm. The collimation was 64 × 0.67 or 128 × 0.625 for DECT and 64 × 0.275 for SPCCT. Bolus tracking was used for DECT, with a 110 Hounsfield Unit (HU) cut-off region of interest (ROI) placed in the descending aorta, while for SPCCT, a bolus test of 20 mL of Iomeprol was first followed by a saline flush of 20 mL.

### CT reconstructions

Images were reconstructed at a specific mid-diastolic phase (78%) of the R–R interval. A small FOV of 220 mm was used, with a matrix size of 512 × 512 mm for DECT and 1,024 × 1,024 for SPCCT. Slice thickness was 0.67 mm for DECT and 0.25 mm for SPCCT. A medium-smooth XCB (Xres cardiac standard) and a Detailed 2 kernel were applied for DECT and SPCCT, respectively. Iterative reconstruction algorithms (4th generation) were applied in both systems, with the use of iDose^4^ level 3 for DECT and level 6 SPCCT (Phillips Healthcare). Conventional, monoenergetic 40 keV, monoenergetic 50 keV and iodine map images were generated for both systems. Only 40 keV and 50 keV virtual monoenergetic images were reconstructed and analysed, as these low-energy levels have been shown in previous studies to provide optimal contrast and iodine signal for myocardial imaging [24–26].

### Reference standard classification of myocardial perfusion status

The reference perfusion status of the myocardial segments (normal, hypoperfused, infarcted) was defined based on a comprehensive evaluation that included both ICA, as well as other imaging and clinical data. Hemodynamically significant coronary stenosis was defined as either a FFR < 0.8, indicating physiologically significant stenosis, or a stenosis severity of > 70% observed on ICA. Segments were categorised as normal if they were FFR-negative (≥ 0.8) or without severe stenosis (< 70%) based on ICA findings. Lesions in the inferior territory were attributed to a specific territory according to the patient’s right or left coronary dominance. Detailed patient records were reviewed, including previous invasive angiography, myocardial scintigraphy, or documentation of prior myocardial infarction, to confirm the absence of infarction or other abnormalities in segments labelled as normal. Segments associated with prior myocardial infarction documented in the clinical history were excluded from the analysis.

### CT image analysis

**Fig. 2:**
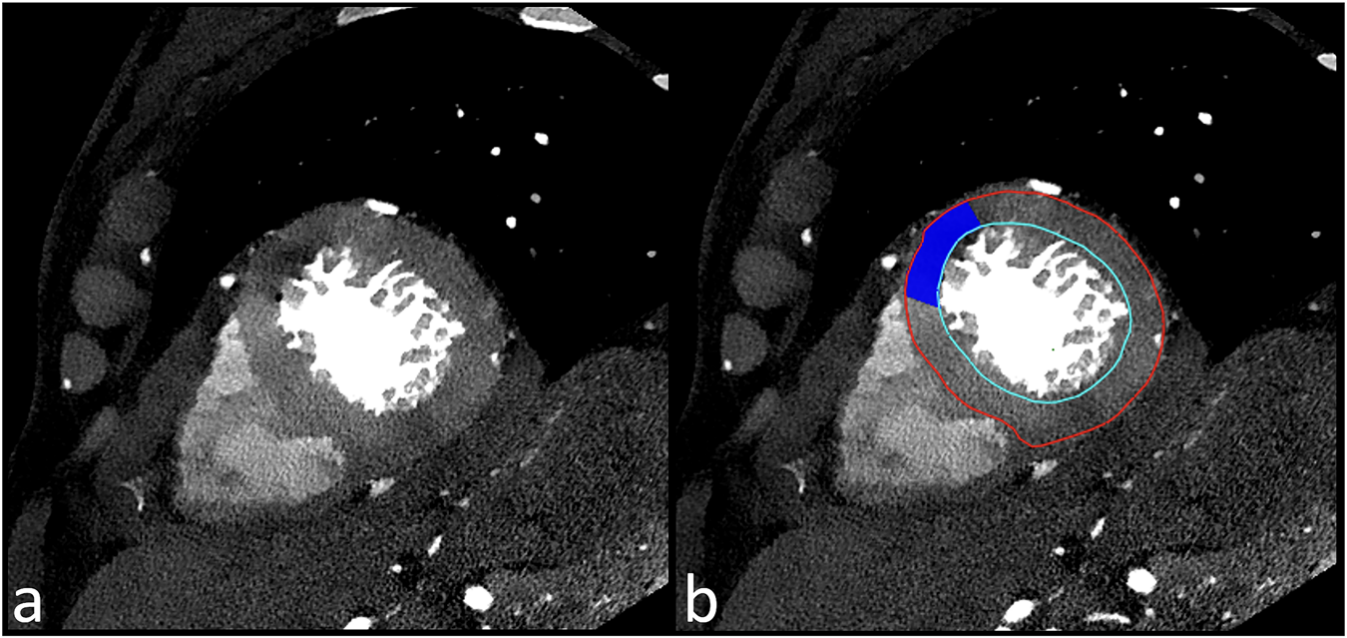
Example of lesion tagging using custom-made software, showing the first-pass myocardial perfusion images in short-axis view without (**a**) and with (**b**) an overlay interface, on SPCCT. A hypoperfusion area in the mid-anterior territory is tagged from endocardium to epicardium (blue area)

Images were displayed and analysed in a dedicated station with an integrated custom-made software with the ability to automatically identify the endocardial and epicardial contours of the left ventricular myocardium. After manual correction of the contours, if necessary, the software could automatically divide the volume into the 17 myocardial segments, according to the American Heart Association recommendations [27]. Images were reviewed independently by two readers (G.F. and S.B.) with 4 years and 11 years of experience in cardiovascular imaging, respectively. Myocardial segments were displayed in a short-axis view with the possibility of changing the plane to the long axis (Fig. 2). All conventional or spectral reconstructions could be selected for visualisation. Each myocardial segment was subdivided into 36 subsegments: 4 concentric layers extending from the endocardium to the epicardium, further divided into 3 zones along the septal-lateral direction and 3 zones along the baso-apical direction of the myocardium. Each subsegment could be selected and subjectively tagged as a region of interest (ROI), with four possible labels: normal, hypoperfusion, infarction or artefacts. Subsegments tagged as impaired due to artefacts such as photon starvation were excluded from analysis. Observers were blind to the history of the patients, to the results of the ICA and did not evaluate coronary arteries on the CT exams.

Quantitative attenuation HU values were collected for each normal and hypoperfused subsegment in both modalities.

### Performance evaluation of the CT systems

Sensitivity, specificity and accuracy were assessed for all three coronary artery territories, as well as for all territories, both for DECT and SPCCT. The coronary artery territories were defined as previously reported [26].

Agreement between reviewers for subsegments tagging was assessed using a weighted (κ) correlation, with values interpreted as: < 0 indicating no agreement, 0.01–0.20 as slight, 0.21–0.40 as fair, 0.41–0.60 as moderate, 0.61–0.80 as substantial, and 0.81–1.00 as almost perfect agreement.

### Statistical analysis

Statistical analysis was performed using RStudio (version 2023.09). Continuous values are displayed as mean ± SD or median [IQR] depending on their distribution. Diagnostic accuracy metrics were derived from the pooled results of both readers. The Student *t*-test was used to compare mean differences. Cohen’s weighted kappa was used to assess inter-observer agreement. 95% confidence intervals were calculated using the Clopper–Pearson method. A *p* < 0.05 was considered statistically significant.

## Results

### Population and coronary stenoses

A total of 18 patients (mean age 63.1 ± 11.5 years, 83% male, mean BMI 27.7 ± 6.3 kg/m²) were analysed, corresponding to 306 myocardial segments. Among them, 15 (83.3%) patients had at least one hemodynamically significant stenosis, while 3 (16.6%) patients had negative FFR findings and no severe stenosis. Out of the 54 coronary arteries involved, 15 (27.7%) presented a hemodynamically significant stenosis, of which 11 (73.3%) with a severe (> 70%) stenosis and 4 (26.6%) with an FFR-positive stenosis. Notably, most lesions with severe stenosis (*n* = 11, 73%) were lesions with > 80% stenosis. The territories of 15 (27.7%) coronary arteries were excluded due to clinically documented myocardial infarction. Of the hemodynamically significant stenoses, 8 (53.3%) involved the left descending artery, 3 (20.0%) the circumflex artery and 4 (26.6%) the right coronary artery (RCA). Patients’ and stenoses’ characteristics are shown in Table 1.

**Table 1:** Patients’ and lesions’ characteristics

Parameter	Value
General characteristics	
Patients (*n*)	18
Men (*n*)	15 (83.3%)
Women (*n*)	3 (16.6%)
Age (yo)	63.1 ± 11.5
Weight (kg)	80.2 ± 23.5
Size (cm)	170.3 ± 8.4
BMI (kg/m^2^)	27.7 ± 6.3
Coronary artery disease	
Coronary stenoses (*n*)	15
FFR positive stenoses (*n*)	4 (26.6%)
Severe (> 70%) stenoses (*n*)	11 (73.3%)
Left anterior descending artery stenoses (*n*)	8 (53.3%)
Circumflex artery stenoses (*n*)	3 (20.0%)
RCA stenoses (*n*)	4 (26.6%)

If not specified otherwise, data expressed as means ± SD

*FFR* Fractional flow reserve, *BMI* Body mass index

### CT characteristics of the myocardium

Out of the 612 segments (306 assessed myocardial segments overall x the two observers), 511 (83.5%) were tagged as normal on DECT and 479 (78%) were tagged as normal on SPCCT. A total of 52 (8.5%) segments were tagged as hypoperfused on DECT, including 35 (67.3%) on the left anterior descending (LAD) artery territory, 8 (15.3%) on the left circumflex (LCX) artery territory and 9 (17.3%) on the RCA territory. For SPCCT, a total of 68 (11.1%) segments were tagged as hypoperfused, including 52 (76.4%) on the left anterior descending artery (LAD) territory, 9 (13.2%) on the circumflex coronary artery territory and 7 (10.3%) on the RCA territory. Forty-nine (8.0%) segments were tagged as artefacts on DECT, while 65 (10.6%) segments were tagged as artefacts on SPCCT. For DECT, artefacts were distributed as follows: 8 (16.3%) in the LAD territory, 21 (42.8%) in the LCX territory, and 20 (40.8%) in the RCA territory. Similarly, for SPCCT, artefacts were distributed as follows: 7 (10.7%) in the LAD territory, 31 segments (47.7%) in the LCX territory, and 27 segments (41.5%) in the RCA territory.

**Fig. 3:**
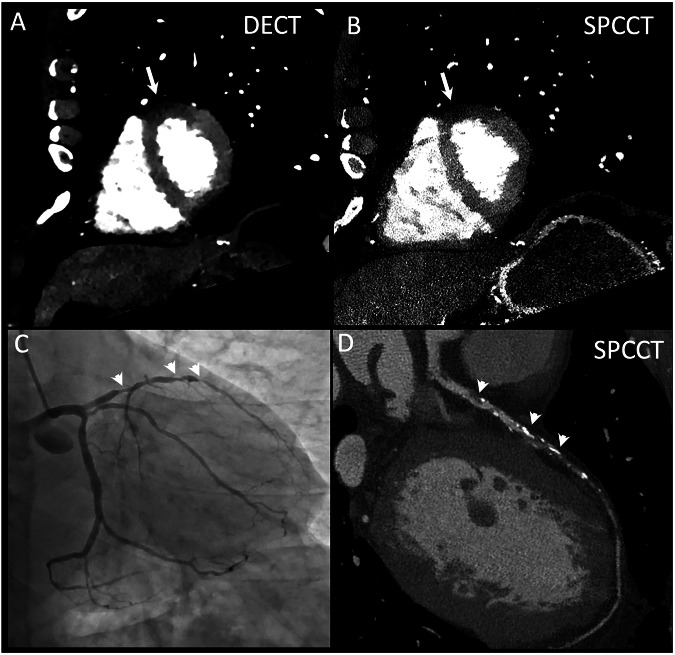
Short-axis view of DECT (**A**) and SPCCT (**B**) first-pass MPI images showing hypoperfusion areas (arrows) in a 61-year-old male with severe (> 80%) stenoses (arrowheads) of the LAD, as seen on ICA (**C**) and SPCCT (**D**). Perfusion images are displayed at 40 keV monoenergetic reconstruction

An example of normal and hypoperfused territories in both modalities is shown in Fig. 3. The mean attenuation of the myocardium on conventional images was 89.5 ± 35.4 HU for DECT and 84.1 ± 80.3 HU for SPCCT. On conventional images, the mean attenuation of the normal segments was 97.1 ± 43.9 HU for DECT and 91.3 ± 86.7 HU for SPCCT, while the mean attenuation of the hypoperfused segments was 79.2 ± 35.2 HU for DECT and 75.5 ± 74.8 HU for SPCCT. This resulted in a mean difference between normal and hypoperfused segments of 17.9 HU for DECT (95% CI: 8.1–27.7) and 15.8 HU for SPCCT (95% CI: -8.4 to 40.0), both with *p* < 0.05. Attenuation results are summarised in Table 2.

**Table 2:** Quantitative analysis of myocardial attenuation

	DECT	SPCCT
Mean attenuation of myocardium	89.5 ± 35.4	84.3 ± 80.3
Mean attenuation of normal segments	97.1 ± 43.9	91.3 ± 86.7
Mean attenuation of hypoperfused segments	79.2 ± 35.2	75.5 ± 74.8
Mean difference (normal *versus* hypoperfused)	17.9 ± 20.2	15.8 ± 45.2
95% CI for mean difference (HU)	8.1–27.7	-8.4–40.0

Results are displayed as HU ± standard deviation

*DECT* Dual-energy CT, *SPCCT* Spectral photon-counting CT, *CI* Confidence Intervals

### Performance evaluation of the CT systems

Overall, DECT achieved a sensitivity of 73.3% a specificity of 75% and an accuracy of 74.4% while SPCCT achieved a sensitivity of 73.3%, a specificity of 79.2%, and an accuracy of 76.9%.

For the LAD territory, SPCCT demonstrated better performance with a sensitivity of 87.5%, a specificity of 100%, and an accuracy of 90%. In comparison, DECT achieved a sensitivity of 62.5%, a specificity of 50%, and an accuracy of 60%, reflecting its lower diagnostic accuracy for this critical territory.

In the LCX territory, DECT achieved the highest sensitivity across all territories at 100%, along with a specificity of 90.0% and an accuracy of 92.3%. Conversely, SPCCT performed less favourably in this territory, with a sensitivity of 66.7%, a specificity of 80%, and an accuracy of 76.9%.

For the RCA territory, both modalities showed comparable accuracy of 68.8%. However, DECT had a higher sensitivity (75.0% *versus* 50%), while SPCCT demonstrated better specificity (75.0% *versus* 66.7%).

**Fig. 4:**
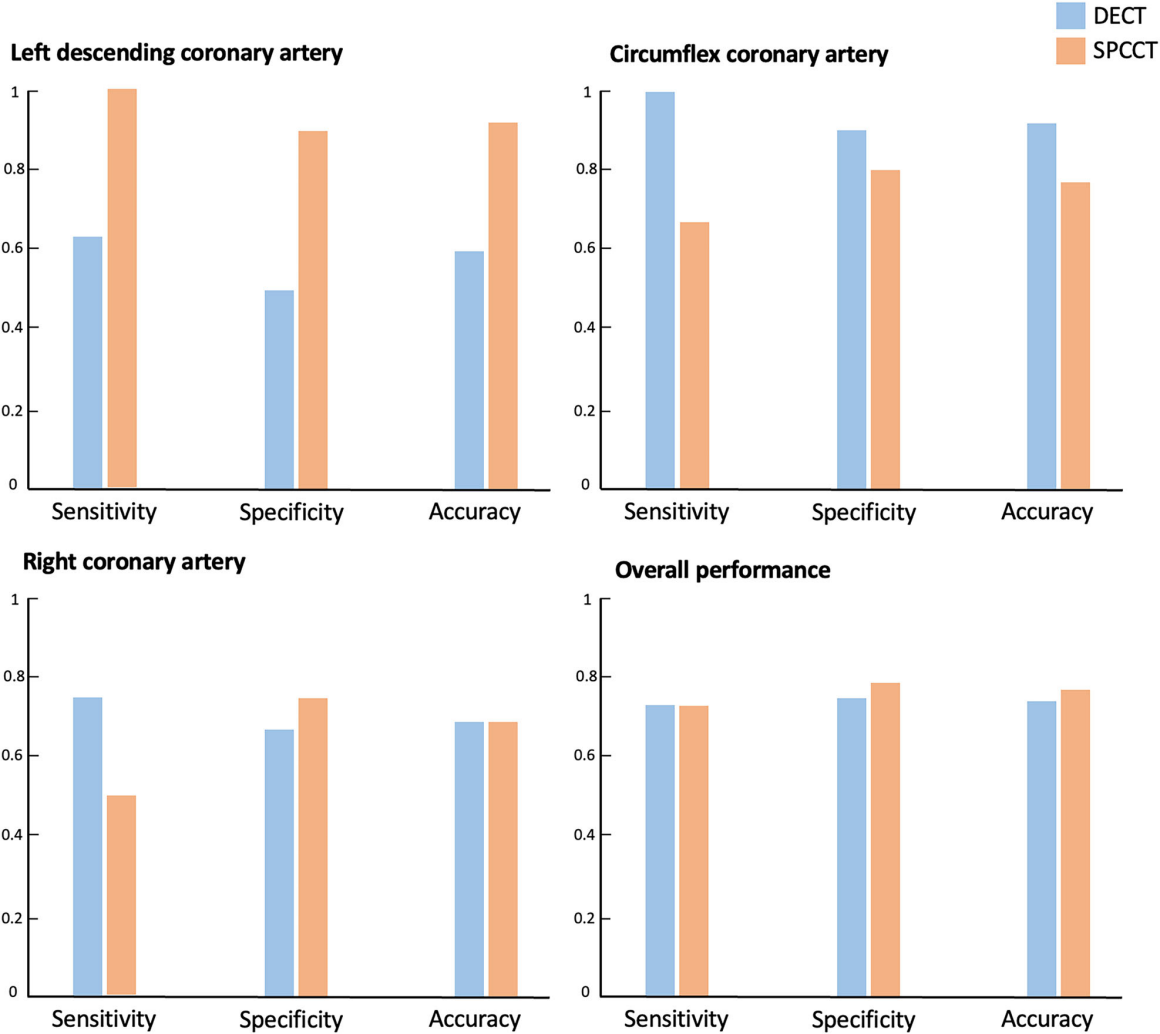
Performance of DECT and SPCCT in detecting hypoperfused territories, including sensitivity, specificity, and accuracy. Results are shown for the three main coronary arteries and all detected lesions. DECT, Dual-energy CT; SPCCT, Spectral photon-counting CT

Performance results are summarised in Fig. 4 and Table 3.

**Table 3:** Performance metrics of DECT and SPCCT for MPI across coronary artery territories and overall

	Sensitivity (%)	Specificity (%)	PPV (%)	NPV (%)	Accuracy (%)
DECT					
LAD	62.5 (24.5–91.5) [5/8]	50.0 (1.3–98.7) [1/2]	83.3 (35.9–99.6) [5/6]	25.0 (0.6–80.6) [1/4]	60.0 (26.2–87.8) [6/10]
Circumflex artery	100 (29.2–100) [3/3]	90.0 (55.5–99.7) [9/10]	75.0 (19.4–99.4) [3/4]	100 (66.4–100) [9/9]	92.3 (64.0–99.8) [12/13]
RCA	75.0 (19.4–99.4) [3/4]	66.7 (34.9–90.1) [8/12]	42.9 (9.9–81.6) [3/7]	88.9 (51.8–99.7) [8/9]	68.8 (41.3–89.0) [11/16]
Overall (DECT)	73.3 (44.9–92.2) [11/15]	75.0 (53.3–90.2) [18/24]	64.7 (38.3–85.8) [11/17]	81.8 (59.7–94.8) [18/22]	74.4 (57.9–87.0) [29/39]
SPCCT					
LAD	87.5 (47.3–99.7) [7/8]	100 (15.8–100) [2/2]	100 (59–100) [7/7]	66.7 (9.4–99.2) [2/3]	90.0 (55.5–99.7) [9/10]
Circumflex artery	66.7 (9.4–99.2) [2/3]	80.0 (44.4–97.5) [8/10]	50.0 (6.8–93.2) [2/4]	88.9 (51.8–99.7) [8/9]	76.9 (46.2–95.0) [10/13]
RCA	50.0 (6.8–93.2) [2/4]	75.0 (42.8–94.5) [9/12]	40.0 (5.3–85.3) [2/5]	81.8 (48.2–97.7) [9/11]	68.8 (41.3–89.0) [11/16]
Overall (SPCCT)	73.3 (44.9–92.2) [11/15]	79.2 (57.8–92.9) [19/24]	68.8 (41.3–89.0) [11/16]	82.6 (61.2–95.0) [19/23]	76.9 (60.7–88.9) [30/39]

95% Confidence intervals are shown in parentheses; raw values are shown in square brackets

*DECT* Dual-energy CT, *SPCCT* Spectral photon-counting CT, *PPV* Positive predictive value, *NPV* Negative predictive value

### Inter-observer agreement

Overall, the agreement between reviewers was substantial, with a κ correlation of 0.75 (95% CI: 0.62–0.88, *p* < 0.001), corresponding to an observed agreement of 85% and a disagreement rate of 15%. When analysing each modality separately, DECT showed substantial agreement overall (κ = 0.62, 95% CI: 0.45–0.79, *p* < 0.001; observed agreement 78%, disagreement 22%), while SPCCT demonstrated better, almost perfect agreement overall (κ = 0.86, 95% CI: 0.75–0.97, *p* < 0.001; observed agreement 93%, disagreement 7%). For DECT, agreement was substantial for the LAD territory (κ = 0.73, 95% CI: 0.56–0.90, *p* < 0.001; observed agreement 88%), substantial for the LCX territory (κ = 0.72, 95% CI: 0.40–1.00, *p* = 0.146; observed agreement 85%), and fair for the RCA territory (κ = 0.38, 95% CI: 0.15–0.61, *p* < 0.001; observed agreement 65%). For SPCCT, agreement was substantial to perfect across all territories: LAD (κ = 0.73, 95% CI: 0.57–0.89, *p* < 0.001; observed agreement 88%), LCX (κ = 0.87, 95% CI: 0.75–0.99, *p* < 0.001; observed agreement 92%), and RCA (κ = 0.93, 95% CI: 0.85–1.00, *p* < 0.001; observed agreement 95%).

## Discussion

This study sought to evaluate the comparative performance of DECT and SPCCT for MPI at rest in a very high-risk population with confirmed hemodynamically significant coronary stenoses. Notably, we demonstrated that hypoperfusion defects in the territory of coronary arteries presenting severe stenosis can be detected on rest images using both modalities, highlighting their potential for non-invasive myocardial assessment in this challenging patient group.

While the comparison between SPCCT and DECT in cardiac imaging has been explored to some extent in coronary artery analysis [17, 28–32], the evaluation of myocardial perfusion remains relatively under-investigated, especially with SPCCT. Previously, Sánchez-Gracián et al [33] explored myocardial analysis using a dynamic perfusion protocol with DECT, showing promising results for assessing myocardial tissue differentiation. Van Assen et al [9] demonstrated that DECT iodine quantification differentiates normal, ischaemic, and infarcted myocardium on first-pass images, with iodine levels significantly varying across these tissue types. More recently, iodine maps and iodine quantification have been explored in DECT at rest. D’angelo et al [10] showed that spectral data in CCTA can improve diagnostic accuracy by assessing myocardial iodine distribution, reflecting coronary perfusion even at rest. Notwithstanding these promising results, myocardial perfusion with DECT is subject to imperfections and variabilities such as a dependency on the injection protocol, as demonstrated by Boccalini et al in patients without significant stenosis [26]. Most studies on the assessment of the myocardium with photon counting CT so far have focused on the analysis of late-enhancement acquisitions [34–36]. For instance, Aquino et al [37] investigated the comparison of extracellular volume quantification between SPCCT and MRI, highlighting the potential of SPCCT as an alternative imaging modality for myocardial tissue characterisation.

Our findings indicate that SPCCT offers overall comparable performance to DECT. SPCCT outperformed DECT in the LAD territory, which is particularly important given that the majority of stenoses were located in this region. The results of other coronary territories should be regarded with caution due to the limited number of assessed stenosis, and further studies are needed to confirm our findings. Inclusion of a control population without significant coronary stenoses would have strengthened the study, particularly for estimating the rate of false positives. However, our protocol involved two CT exams and one ICA, making it ethically unjustifiable for low-risk or healthy individuals. In our institution, ICA with FFR is typically reserved for very high-risk patients with a high atherosclerotic burden, which led to the selection of this specific patient population. Unfortunately, due to their novelty, our results cannot be directly compared to data from the literature. Nevertheless, they seem particularly promising also in light of the absence of additional injection, radiation dose, acquisition, external analysis, or exam for the patients, otherwise necessary for other established modalities such as dynamic perfusion, stress perfusion, or FFR-CT [38].

We found a predominance of artefacts in the circumflex artery (LCX) territory for both DECT and SPCCT. This finding can be attributed to known challenges specific to this region, including motion artefacts at the base of the heart, beam-hardening effects caused by the proximity of the opacified descending thoracic aorta in the arterial phase and other causes [26, 33]. These challenges remain evident for the clinical prototype SPCCT, which is expected to show improvements in the future.

The quantitative analysis of myocardial attenuation revealed similar results between DECT and SPCCT, with mean attenuation differences of 17 HU and 16 HU between normal and hypoperfused segments, respectively. This indicates that both modalities can provide images with visually detectable differences between hypoperfused and normal segments. In our study, we analysed the images without restriction on the type of images used, whether conventional, monoenergetic, or iodine images, so that the performance of specific reconstructions was not evaluated, highlighting an area with potential for future studies. For example, Gnasso et al [39] recently explored monoenergetic ranges for the assessment of diffuse myocardial fibrosis through the quantification of extracellular volume with SPCCT and concluded that a 45 keV monoenergetic reconstruction provided the best performance. Furthermore, in our study, we used very thin slices with SPCCT, which would have maximised the spatial resolution for an eventual concomitant coronary artery assessment. Indeed, the superiority of SPCCT for coronary artery assessment relies on the exploitation of its ultra-high resolution [17, 40]. This choice was meant to highlight the fact that both analyses are possible on data from one single acquisition with SPCCT, since it always allows for spectral reconstructions. This is similar to what is possible with detector-based DECT systems, such as the ones we used in this study. On the contrary, source-based DECT systems are different since a dual-energy CT acquisition mode needs to be selected beforehand and performed with parameters that might not be optimised for coronary artery assessment [41, 42]. Furthermore, our choice of reconstruction parameters also took into account data storage considerations, as SPCCT ultra-high resolution images can generate large data volumes that may challenge PACS capacity [15]. While improved hospital servers may mitigate these concerns, a single reconstruction enabling assessment of both coronary arteries and myocardium on the same images remains a practical advantage. However, we did not explore other reconstruction types, such as those with thicker slices, which could potentially impact lesion detection. Additionally, the classification of coronary artery territories based on a standard model may not capture the individual patient variations, such as perfusion of a territory by another branch, highlighting this variability as a possible direction for future research.

Reader agreement was consistent, with substantial agreement for both DECT and SPCCT. SPCCT demonstrated stronger agreement (κ = 0.86) compared to DECT (κ = 0.62). Despite the higher κ value for SPCCT, this difference may not be clinically significant and could be due to random variation.

This study is subject to limitations. First, the small sample size, particularly the limited number of patients with hemodynamically significant lesions, restricts the generalizability of the findings. The low number of lesions in the circumflex and right coronary arteries limits the interpretability of results between DECT and SPCCT for these territories. Second, the very high-risk nature of the patient population presents inherent challenges for image acquisition. Suboptimal image quality may have occurred due to poor cardiac function, leading to suboptimal iodine injection. The complexity of these patients could have influenced the overall performance of both imaging modalities. For example, the marked variability in attenuation values limits the ability to define clear thresholds for hypoperfusion. Moreover, based on data from the literature, most 70–80% stenoses will result in hypoperfusion at rest, so that these values are broadly considered as a threshold [2, 43, 44]. Nevertheless, in a minority of cases, this might not hold true. Additionally, the absence of an independent perfusion imaging comparator prevents direct validation of the hypoperfusion defects identified by SPCCT and DECT. Third, while SPCCT has a detector coverage of less than 2 cm, potentially perceived as a disadvantage compared to the 4–8 cm coverage available in DECT for this type of analysis, this limitation did not appear to affect the outcomes in our study. Additionally, inherent differences in reconstruction software, kernel parameters, and spatial resolution between the DECT and SPCCT systems may have influenced image quality and diagnostic performance. Fourth, reader awareness of the high-risk nature of the population may have increased sensitivity, despite the observers being blind to the number and location of the lesions, but also introduced false positives. Last but not least, we did not perform any late enhancement acquisition, nor any other imaging exam dedicated to the detection of infarcted areas, so that myocardial infarctions without CT-detectable remodelling and that were not highlighted by previous exams, or the clinical history, might have remained unnoticed.

## Conclusion

SPCCT and DECT provided similar diagnostic performance for rest MPI in a very high-risk population, suggesting that both modalities might effectively detect the hemodynamic consequence of severe coronary artery stenoses.

## Data Availability

Data generated or analysed during the study are available from the corresponding author on reasonable request.

## Abbreviations

CCTA: Coronary computed tomography angiography
DECT: Dual-energy computed tomography
FFR: Fractional flow reserve
LAD: Left anterior descending artery
LCX: Left circumflex artery
MPI: Myocardial perfusion imaging
RCA: Right coronary artery
SPCCT: Spectral photon counting computed tomography
